# Mortality and morbidity following exercise-based renal rehabilitation in patients with chronic kidney disease: the effect of programme completion and change in exercise capacity

**DOI:** 10.1093/ndt/gfy351

**Published:** 2018-11-30

**Authors:** Sharlene A Greenwood, Ellen Castle, Herolin Lindup, Juliet Mayes, Iain Waite, Denise Grant, Emmanuel Mangahis, Olivia Crabb, Kamer Shevket, Iain C Macdougall, Helen L MacLaughlin

**Affiliations:** 1 Department of Therapies, King’s College Hospital, London, UK; 2 Department of Renal Medicine, King’s College Hospital, London, UK; 3 Renal Sciences, Department of Transplantation, Immunology and Mucosal Biology, King’s College London University, London, UK; 4 Department of Nutrition and Dietetics, King’s College Hospital, London, UK; 5 Department of Nutritional Sciences, School of Life Sciences, King’s College London University, London, UK

**Keywords:** chronic kidney disease, morbidity, mortality, rehabilitation, survival

## Abstract

**Background:**

Twelve weeks of renal rehabilitation (RR) have been shown to improve exercise capacity in patients with chronic kidney disease (CKD); however, survival following RR has not been examined.

**Methods:**

This study included a retrospective longitudinal analysis of clinical service outcomes. Programme completion and improvement in exercise capacity, characterised as change in incremental shuttle walk test (ISWT), were analysed with Kaplan–Meier survival analyses to predict risk of a combined event including death, cerebrovascular accident, myocardial infarction and hospitalisation for heart failure in a cohort of patients with CKD. Time to combined event was examined with Kaplan–Meier plots and log rank test between ‘completers’ (attended >50% planned sessions) and ‘non-completers’. In completers, time to combined event was examined between ‘improvers’ (≥50 m increase ISWT) and ‘non-improvers’ (<50 m increase). Differences in time to combined event were investigated with Cox proportional hazards models (adjusted for baseline kidney function, body mass index, diabetes, age, gender, ethnicity, baseline ISWT and smoking status).

**Results:**

In all, 757 patients (male 54%) (242 haemodialysis patients, 221 kidney transplant recipients, 43 peritoneal dialysis patients, 251 non-dialysis CKD patients) were referred for RR between 2005 and 2017. There were 193 events (136 deaths) during the follow-up period (median 34 months). A total of 43% of referrals were classified as ‘completers’, and time to event was significantly greater when compared with ‘non-completers’ (P = 0.009). Responding to RR was associated with improved event-free survival time (P = 0.02) with Kaplan–Meier analyses and log rank test. On multivariate analysis, completing RR contributed significantly to the minimal explanatory model relating clinical variables to the combined event (overall χ^2^ = 38.0, P* *<* *0.001). ‘Non-completers’ of RR had a 1.6-fold [hazard ratio = 1.6; 95% confidence interval (CI) 1.00–2.58] greater risk of a combined event (P* *=* *0.048). Change in ISWT of >50 m contributed significantly to the minimal explanatory model relating clinical variables to mortality and morbidity (overall χ^2^ = 54.0, P* *<* *0.001). ‘Improvers’ had a 40% (hazard ratio = 0.6; 95% CI 0.36–0.98) independent lower risk of a combined event (P* *=* *0.041).

**Conclusions:**

There is an association between completion of an RR programme, and also RR success, and a lower risk of a combined event in this observational study. RR interventions to improve exercise capacity in patients with CKD may reduce risk of morbidity and mortality, and a pragmatic randomised controlled intervention trial is warranted.

## ADDITIONAL CONTENT

An author video to accompany this article is available at: https://academic.oup.com/ndt/pages/author_videos.

## INTRODUCTION

Cardiovascular disease (CVD) is the leading cause of morbidity and mortality in patients with chronic kidney disease (CKD). Although many patients with CKD have other risk factors for CVD (e.g. diabetes, smoking, sedentary lifestyle, hypertension), and part of the increased risk is attributable to these risk factors, studies demonstrate that CKD itself is a major independent risk factor [1]. As renal function declines, the association with CVD increases, and patients with non-dialysis-requiring CKD are more likely to die from CVD than to develop end-stage renal disease. There is evidence of an increased prevalence of physical symptoms associated with declining kidney function [2]. Exercise-based rehabilitation, which promotes a more physically active lifestyle, has the potential to positively impact upon functional ability, aerobic capacity and the quality of life of patients with CKD, independent of the stage of the disease process [3–5]. Despite published recommendations calling for physical activity (PA) and exercise counselling for patients with CKD [6, 7], exercise-based renal rehabilitation (RR) for patients with CKD is not routinely offered to patients.

Cardiorespiratory capacity, as measured by the integrated index of peak oxygen uptake, has been identified as prognostically important for CVD and all-cause mortality in the general population [8], and in patients with CKD Stage 5 [9]. Habitual levels of PA, as measured using self-report questionnaires, are also linked to CV health. Physical inactivity has been shown to be a strong independent risk factor for CV morbidity and mortality in patients with CKD Stage 5 [10] and in patients with CKD Stages 2–4 [11, 12]. Physical inactivity, physical function limitations, muscle mass and muscle function-related measures have also been identified as strong predictors of disease progression and survival in patients at all stages of CKD [2, 13, 14]. Self-reported physical function, as evaluated using the physical component score from the SF-36 questionnaire, has also been shown to carry a significant hospitalisation and survival prognostic value for patients on dialysis [15, 16]. To our knowledge, no studies have compared the survival rates in patients with CKD who have completed a pragmatic RR exercise-based intervention. A Cochrane review [17], and a systematic review and research evidence synthesis of studies investigating exercise therapy for patients with CKD [18], suggest that as the studies to date have relatively short duration of interventions and follow-up periods, combined with extremely small sample sizes, there has been little opportunity thus far for any real observations with regards to the effect of exercise-based rehabilitation on morbidity and mortality rates in patients with CKD.

The King’s College Hospital RR programme for patients with CKD is a complex exercise-based rehabilitation programme comparable in design to the cardiac rehabilitation (CR) [19, 20] and pulmonary rehabilitation (PR) [21] programmes that are routinely offered to those patients with disease-specific long-term conditions in the UK. A systematic review and meta-analysis showed that those patients after myocardial infarction who attended CR had a lower risk of all-cause mortality than non-attendees [22]. A recent study by Houchen-Wolloff *et al*. [23] suggested that there was an association between the successful completion of PR and survival in patients with chronic obstructive pulmonary disease (COPD), and that PR success [>50 m change in incremental shuttle walk test (ISWT) walking distance] was associated with improved survival in patients with COPD.

In the interest of evaluating whether the RR programme, offered to patients at all stages of the CKD trajectory at King's College Hospital (KCH), was able to offer a comparable event-free survival advantage when compared with that reported for PR in the recent study by Houchen-Wolloff *et al*. [23], we declared the following hypothesis for our study: the successful completion of a pragmatic RR programme (>50%) for patients with CKD and RR programme success (>50 m change in walking distance) would be associated with a longer event-free period of time in patients with CKD. Our aims, similar to those of Houchen-Wolloff *et al*. [23], were to compare the long-term morbidity and mortality rates in two cohorts of patients referred to RR: those who successfully completed RR, and a comparator group constructed from patients who either did not complete RR or did not start the programme. Additionally, we compared survival between those people who were able to achieve a clinically meaningful improvement in ISWT (>50 m walking distance) following RR with those who were not.

## MATERIALS AND METHODS

We conducted a retrospective longitudinal analysis of clinical service outcomes on all patients with a confirmed diagnosis of CKD who had been assessed for outpatient-based RR at King’s College Hospital NHS Trust over a 12-year period from 2005 to 2017. A total of 757 patients (male 54%) from across the CKD trajectory (242 haemodialysis, 221 kidney transplant recipients, 43 peritoneal dialysis, 251 non-dialysis CKD) had predominately been referred to the RR service by nephrologists or members of the renal multidisciplinary team at the hospital. Participants were included for analysis if they had given their consent for their data from the RR assessment to be recorded and evaluated for audit purposes. The only exclusion criteria were missing data that would preclude ascertaining morbidity or mortality status for the individual. The study was considered by Camberwell and St Giles London Research Ethics Committee to be an evaluation of a clinical service and as such, not requiring ethical approval.

All RR measures recorded were part of the clinical care of patients referred to the RR service, and were recorded by a trained health care professional at the initial assessment for RR and reassessed following completion of the programme. Maximal exercise capacity was assessed using the ISWT [24]. For the ISWT, the participants were required to walk around two cones set 9 m apart (so the final track was 10 m) in time to a set of progressive auditory beeps played on a CD. During exercise testing, resting and post-exercise oxygen saturation and heart rate were recorded. The test was concluded when the patient was >0.5 m away from the cone when the beep sounded (allowing one lap to catch up), or if the patient reported that they were too breathless to continue. The ISWT was performed on at least two occasions, 30 min apart, at baseline to reduce any learning effect. Age and gender were recorded together with stage of CKD, smoking history, height and weight, blood pressure and diabetes status. The RR programme was modelled on conventional CR, and aimed to deliver an individualised rehabilitation programme incorporating combined exercise training and self-management education. The RR programme has been described previously [3]. Briefly, it is a comprehensive intervention designed to improve activities of daily living-related functional capacity, reduce symptoms of fatigue and increase motivation, confidence, functional status and health-related quality of life in patients with CKD. A team consisting of a lead renal physiotherapist, a specialist physiotherapist and a technical instructor delivered the exercise, education and self-management advice for the programme. The patients were required to attend twice-weekly supervised outpatient exercise and the education sessions, and to perform once-weekly home-based exercise for a period of 12 weeks. Data were collected at the first visit (baseline) and at 12 weeks. Following completion of the programme, patients were advised to continue their home exercise programme but there were no formal follow-up arrangements.

As our programme has been running for many years, some patients had been assessed for RR on more than one occasion. In this event, it was decided to use baseline data relating to the first completed RR episode, termed the index assessment, to calculate length of follow-up. For patients who had been assessed more than once but each time had either dropped out of RR or not started the programme at all, we used baseline data relating to the first contact with the RR service to calculate length of follow-up. Mortality status for each identified patient was ascertained by interrogating the hospital renalware database and electronic patient records between 17 and 24 October 2017. If patients were event-free to this date, then the number of days from the date of the index RR assessment to date of censor was calculated. If patients had experienced an event [death, cerebrovascular accident (confirmed stroke), myocardial infarction and hospitalisation for heart failure], then the number of days from date of the index assessment to date of event was calculated.

Patients were classified as having completed a course of RR if they completed the post-RR assessment. The data set was rigorously checked for duplicates to ensure that no patient was classified as both having completed a course of RR and also as a non-completer. For those patients who completed RR, we further subdivided people into those who were classified as having successfully ‘improved’ in exercise capacity by achieving a change in ISWT distance of 50 m or more following RR (rounded up from the value of the minimal clinically important difference) of the ISWT recorded for patients completing a PR programme = 47.5 m) or as a ‘non-improver’ in exercise capacity if the change in ISWT was <50 m [25].

Baseline differences were compared between those patients who completed RR and ‘non-completers’, as well as between ‘improvers’ and ‘non-improvers’ to exercise training using independent *t*-tests for continuous variables, while Chi-square tests were used to compare categorical variables. Event-free time was compared for ‘completers’ and ‘non-completers’ and ‘improvers’ and ‘non-improvers’ graphically using Kaplan–Meier survival analysis. Differences in time to combined event in both groups were analysed with the use of the log rank test. Cox regression analysis was used to generate regression β coefficients (B) for a range of baseline variables (age at assessment, gender, ethnicity, smoking status, diabetes, modality and pre-RR ISWT distance) to determine factors that independently predicted the risk of a combined event. The model was run twice, firstly to include the effect of completion of rehabilitation on survival and secondly to include the value of metre change in ISWT for those patients classified as ‘completers’. Continuous variables are presented as mean (SD) unless otherwise stated. A P < 0.05 was considered to be statistically significant in all analyses. Analysis was carried out using Predictive Analytics Software Statistics version 18 (formerly SPSS, IBM Corporation, Armonk, NY, USA).

## RESULTS

Morbidity and mortality status were ascertained for 757 patients (male 54%) from across the CKD trajectory (242 haemodialysis patients, 221 kidney transplant recipients, 43 peritoneal dialysis patients, 251 non-dialysis CKD patients) who were referred for RR over a 12-year period from 2005 to 2017 and fulfilled the inclusion/exclusion criteria. There were 193 events, including 136 deaths during the follow-up period (median follow-up of 34 months). Table 1 presents demographic data and baseline measures for all patients at the time of the index assessment. Patients were predominately haemodialysis, non-dialysis CKD or kidney transplant recipients. An assessment on completion of a course of RR was ascertained for 43% of the patients in the cohort while data were available for 57% patients who did not start or dropped out of rehabilitation and did not go on to complete a further course of RR. Table 2 shows a comparison of the variables at the index assessment between those patients who completed a full course of RR and those who did not start or dropped out. Patients who did not complete RR were found to be younger (55 years versus 58 years, P = 0.001) than ‘completers’, more likely to be from a black and minority ethnic origin (*n* = 293 versus *n* = 172, P = 0.027), receiving haemodialysis therapy (*n* = 169 versus *n* = 80, P = 0.001) or to have received a kidney transplant (*n* = 121 versus *n* = 89, P = 0.027), and to have a lower baseline ISWT distance (261 m versus 296 m, P < 0.001).

**Table 1:** Patient demographics at baseline RR assessment

Mean (SD) unless otherwise stated	*n* = 757
Age at assessment (years)	56.11 (12.38)
Gender (male/female) (%)	54/46
Modality Non-dialysis CKD (%)	251 (33)
Haemodialysis (%)	242 (32)
Peritoneal dialysis (%)	43 (6)
Kidney transplant (%)	221 (29)
Ethnicity Black British/African/Caribbean (%)	429 (55.6)
Asian (%)	86 (11.0)
White Caucasian (%)	242 (31.2)
eGFR (mL/min/1.73 m²)	30.32 (26.65)
Smoker (yes/no) (%)	86/14
Diabetes (yes/no) (%)	59/41
BMI (kg/m^2^)	30.79 (7.00)
ISWT distance (m)	289.92 (170.69)

eGFR, estimated glomerular filtration rate.

**Table 2:** Baseline variables for those patients who completed and did not complete RR

Baseline mean (SD) unless otherwise stated	Completed RR, *n* = 335 (44.3%)	Did not complete RR, *n* = 422 (55.7%)	P-value
Age at assessment (years)	58.44 (11.90)	54.96 (11.32)	<0.001
Men, *n* (%)	184 (56.1)	226 (53.9)	NS
Women, *n* (%)	151 (43.9)	196 (46.1)	NS
Modality			<0.001
Non-dialysis CKD (%)	138 (44.2)	101 (23.8)	
Haemodialysis (%)	80 (24.4)	169 (40.5)	
Peritoneal dialysis (%)	28 (4.8)	31 (6.8)	
Kidney transplant (%)	89 (26.6)	121 (28.6)	
Ethnicity			0.027
Black British/African/Caribbean (%)	147 (52.9)	244 (57.8)	
Asian (%)	25 (9.0)	49 (11.6)	
White Caucasian (%)	102 (36.7)	117 (27.7)	
BMI (kg/m^2^)	31.32 (6.33)	30.71 (6.00)	NS
eGFR for all modalities (mL/min/1.73 m²)	32.96 (28.32)	27.93 (26.16)	NS
eGFR non-dialysis CKD	40.43 (31.04)	37.47 (29.07)	
eGFR kidney transplant	45.77 (22.64)	47.62 (24.73)	
Diabetes (yes/no) (%)	35.1/64.9	44.7/55.3	0.013
Hypertension (yes/no) (%)	82.9/17.1	82.0/18.0	NS
Smoker (yes/no) (%)	12.9/87.1	12/1/87.9	NS
ISWT (m)	295.68 (162.43)	260.67 (157.57)	<0.001

eGFR, estimated glomerular filtration rate; NS, non-significant.

**FIGURE 1:**
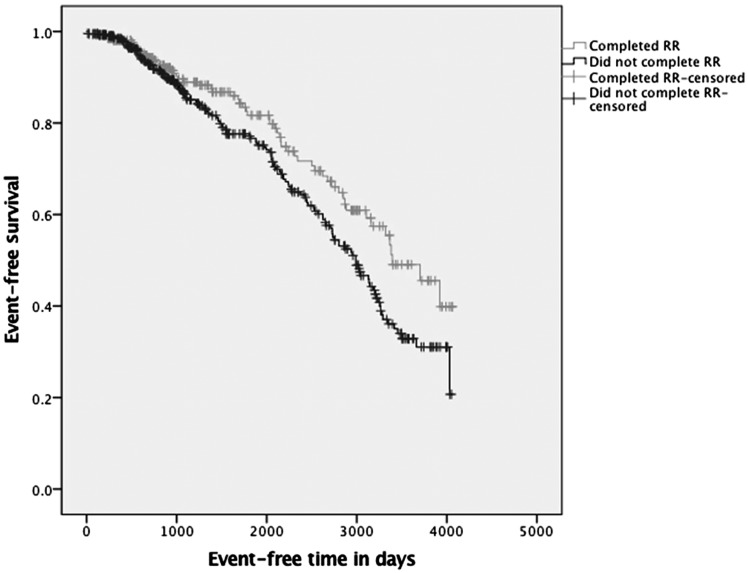
Kaplan–Meier survival analysis for ‘completers’ and ‘non-completers’ of RR.

Figure 1 presents the Kaplan–Meier survival analysis for ‘completers’ and ‘non-completers’ of RR. A statistically significant difference was found between the two groups (log rank test, P = 0.009). Cox regression was used to determine which factors independently predicted time to the combined event. Factors used in the model were baseline age, smoking status, gender, ethnicity, diabetes, body mass index (BMI), modality, baseline ISWT and completion of RR (Table 3). On multivariate analysis, completing an RR programme contributed significantly to the minimal explanatory model relating clinical variables to mortality and CV morbidity (overall χ^2^ = 38.0, P < 0.001). Patients who did not complete the RR programme had a 1.6-fold independent greater risk of a combined event [hazard ratio = 1.6; 95% confidence interval (95% CI) 1.00–2.58; P* *=* *0.048]. Other factors found to independently predict a longer event-free period were higher baseline ISWT distance (P = 0.016), not having diabetes (P = 0.05) and younger age at assessment (P = 0.002). Gender, modality, ethnicity, smoking status and BMI did not independently predict a longer period of time to event.

**Table 3:** Results of Cox regression analysis to identify factors that independently predict event-free survival time, including completion of an RR programme

Variable	B	Exponential (95% CI) for B	P-value
Non-completion of RR	−0.476	1.609 (1.004–2.580)	0.048
Gender	−0.300	0.741 (0.462–1.118)	0.213
Smoker	−0.399	0.671 (0.325–1.384	0.280
BMI	−0.043	0.958 (0.915–1.003)	0.069
Diabetes	−0.512	0.599 (0.359–1.001)	0.050
ISWT (pre-RR)	−0.002	0.998 (0.996–1.000)	0.016
Non-dialysis CKD	0.207	1.231 (0.637–2.376)	0.537
Haemodialysis	−0.370	0.964 (0.553–1.681)	0.686
Peritoneal dialysis	−0.551	0.576 (0.195–1.705)	0.319
Kidney transplant	−0.277	0.758 (0.375–1.532)	0.440
Ethnicity Black British/African/Caribbean	1.005	2.731 (0.885–8.427)	0.081
Asian	0.221	1.248 (0.751–2.074)	0.393
White Caucasian	0.280	0.756 (0.352–1.627)	0.472

**FIGURE 2:**
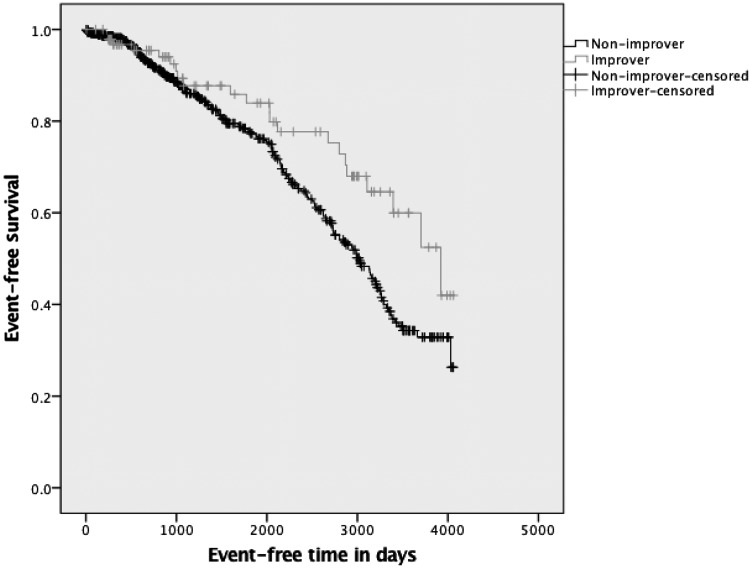
Kaplan–Meier survival analysis for ‘improvers’ and ‘non-improvers’ in exercise capacity.

Of the 335 patients who completed rehabilitation, an increase of 50 m in the ISWT was found in 60% (*n* = 200) of the patients, classified as successful ‘improvers’ in exercise capacity. Figure 2 presents the survival analysis between the ‘improvers’ and ‘non-improvers’ in exercise capacity, and a statistically significant difference in survival time was found between the groups (log rank test, P = 0.02). For those people who completed RR, a second Cox regression model was run using the same factors as described previously, but substituting magnitude of change in ISWT distance instead of completion of RR in the model (Table 4). Change in ISWT of >50 m contributed significantly to the minimal explanatory model relating clinical variables to mortality and CV morbidity (overall χ^2^ = 54.0, P* *<* *0.001). ‘Improvers’ had a 40% independent lower risk of a combined event (hazard ratio = 0.6; 95% CI 0.36–0.98; P* *=* *0.04). A higher baseline ISWT distance (P = 0.001) and younger age (P = 0.019) also predicted a longer event-free period of time.

**Table 4:** Results of Cox regression analysis to identify factors that independently predict event-free survival time, including magnitude of change in exercise capacity (ΔISWT), for patients who completed RR

Variable	B	Exponential (95% CI) for B	P-value
ΔISWT (pre- to post-RR)	−0.522	0.593 (0.360–0.970)	0.041
Age at assessment	0.020	1.020 (1.003–1.038)	0.019
Gender	−0.322	0.718 (0.466–1.105)	0.132
Smoker	−0.396	0.673 (0.341–1.329)	0.254
BMI	−0.006	0.994 (0.982–0.999)	0.313
Diabetes	−0.463	0.629 (0.399–0.991)	0.053
ISWT (pre-RR)	−0.003	0.997 (0.997–0.999)	0.001
Non-dialysis CKD	0.165	1.179 (0.614–2.264)	0.620
Haemodialysis	1.303	1.353 (0.827–2.216)	0.229
Peritoneal dialysis	−0.229	0.795 (0.338–1.872)	0.600
Kidney transplant	−0.242	0.785 (0.338–1.872)	0.600
Ethnicity Black British/African/Caribbean	0.400	1.492 (0.395–3.741)	0.393
Asian	0.019	1.019 (0.656–1.584)	0.932
White Caucasian	−0.507	0.602 (0.305–1.189)	0.144

## DISCUSSION

This study examined the effect of completing an exercise-based RR programme, and also the success in the programme, on the time to the combined outcome of all-cause mortality and CV morbidity in a population of patients with all stages of CKD. There is strong evidence that patients who completed the programme were significantly more likely to have a longer combined event-free period than those who did not complete the programme, along with better baseline exercise capacity (ISWT walking distance), absence of diabetes and younger age. There was also strong evidence that patients who improved their exercise capacity as a result of the exercise programme (achieved a walking distance of >50 m in the ISWT) also were significantly more likely to have a longer combined event-free period than those who did not improve their exercise capacity, along with better baseline exercise capacity and younger age. To our knowledge, this is the first study to report on the association between participation in a disease-specific RR programme and the combined outcome of mortality and CV morbidity in patients with CKD.

The results from our study are in agreement with the results from the study by Houchen-Wolloff *et al*. [23], which reported that patients with COPD who successfully completed a course of PR had a statistically significant survival advantage compared with those patients who dropped out of the programme or failed to start in the first place. The results from our current study similarly demonstrated a significantly longer event-free survival period between those patients who improved by 50 m (or greater) in the ISWT and those who did not. The results suggest that participation in a disease-specific exercise-based rehabilitation programme may be as beneficial for patients with CKD as it is for those patients with COPD who participate in a PR programme [23].

The results of this current study suggest that there is a longer event-free survival period for patients with CKD who complete an RR programme. The RR programme is a complex intervention that is designed to ‘kick-start’ a healthy lifestyle change for patients with CKD. In addition to individualised exercise prescription, there is also a behavioural change and educational component to the programme. The educational component includes discussions on healthy eating, medication usage, living with a long-term condition, self-efficacy for exercise behaviour and increasing and maintaining motivation with PA [3]. It may be suggested that it is this holistic approach that will have contributed to the CV protective effect of completing an RR programme. Our group previously demonstrated that participation in a weight loss programme, which utilised a combination of a low-fat, energy-reduced renal diet, regular exercise, the use of the anti-obesity medication Orlistat (120 mg three times daily, Xenical; Roche Products, Basel, Switzerland) and behavioural therapy techniques to address barriers to lifestyle change, was associated with a longer event-free survival period in patients with CKD, rather than just weight loss alone [26]. The authors concluded that changes in eating habits and PA may have provided greater CV protection than weight loss alone. Our current study supports this hypothesis as we demonstrated that the event-free survival period increased with an improvement in ISWT walking distance. This strongly suggests that an improvement in exercise capacity is a key protective factor against CV morbidity and mortality in patients who complete an RR programme, and supports the evidence demonstrating a link between improved cardio-respiratory fitness, CVD health and survival in patients with CKD [9, 10, 27].

There has been much controversy in recent years around the effectiveness of the pragmatic delivery of exercise-based rehabilitation, when compared with research-specific interventions. This has been particularly pertinent in CR [28, 29]. The 2016 Cochrane review and meta-analysis of randomised controlled trials of CR reported a lack of reduction in mortality [22], in comparison with the previous 2011 review, which reported a reduction in overall mortality, absolute risk reduction, number needed to treat, as well as CV mortality. The inclusion of a pragmatic UK CR trial has been cited as the main reason for this lack of effect on mortality [30]. It would appear reasonable to speculate that the overall dose of exercise prescribed in the pragmatic study was not sufficient or effective in favourably modifying CV risk factors, and thus event-free survival. It is imperative, especially considering the associated link between improved exercise capacity and event-free survival suggested by the results of our current study, that any future pragmatic RR studies aim to accurately quantify and prescribe exercise dosage to improve exercise capacity.

The major limitation of the analysis is the unmeasured confounding, namely that patients who are most motivated to complete an RR course are more motivated to look after themselves, and will therefore live longer. Likewise, patients who have managed to increase their ISWT could be those who are more physically fit, and will therefore live longer. Although we have included these variables in our multivariable risk model, it remains impossible to fully adjust for these confounders without conducting a randomised controlled clinical trial. An additional limitation is that this study is an observation of outcomes of clinical practice. As in the study by Houchen-Wolloff *et al*. [23], we constructed two comparative groups: one made up of people who, for whatever reason, failed to complete RR (or did not start the programme) and the other made up of people who had completed a course of RR. In cases where multiple courses of RR were completed by a single patient, we chose the first complete episode over any subsequent episodes. Although the exercise training offered in the RR classes was encouraged at a moderate level of intensity (modulated with the rate of perceived exertion RPE scoring measure and heart rate monitors), this was not recorded for individual participants. A future randomised controlled trial (RCT) would collect data on the average intensity and duration of exercise sessions and also question whether those participants who exercise at higher intensities, or for longer durations, have fewer events. It is also acknowledged that survival of someone assessed in 2005 may be different from those assessed in 2017 due to advances in the field, and changes in medical treatment. This current study does not report statin usage or albuminuria, which has been found to contribute to mortality risk in patients with CKD [31, 32]. The current study also does not include blood pressure data. Although this was collected as a clinical measure to inform patient safety for exercise training, it was not collected in research conditions.

It is also acknowledged that, in a similar way to the study by Houchen-Wolloff *et al*. [23], we have ‘missed’ a group of patients: those who were unwilling to even be referred for rehabilitation. Event-free survival time in these patients is unknown due to study selection bias. There are a number of additional factors that may have confounded results, and are similar to those cited in our previous work [26]. These include factors such as ability for patients to travel to the hospital to take part in the in-centre RR programme, social support, socio-economic status, literacy and education level. As our study was conducted in a single centre, the results may also not be generalisable to the wider population of patients with CKD. A randomised controlled trial, where all these factors are equal across groups, would be required to fully elucidate whether the relationship we have found in our current study is due to an unmeasured variable, such as motivation to increase exercise and PA levels.

Completion of an RR programme was associated with a longer event-free survival period in our study population. In addition, RR success (>50 m increase in ISWT distance) was associated with a lower risk of a combined event. RR interventions to improve exercise capacity in patients with CKD may reduce the combined risk of CV morbidity and mortality, and a pragmatic randomised controlled intervention trial is warranted.

## FUNDING

S.A.G. is funded by a National Institute for Health Research/Health Education England (NIHR/HEE) Clinical Lectureship. The views expressed are those of the author(s) and not necessarily those of the National Health Service (NHS), the NIHR or the Department of Health.

## AUTHORS’ CONTRIBUTIONS

S.A.G., E.C., J.M., H.L., D.G., E.M., I.W., H.L.M. and I.C.M. contributed to research idea and study design; S.A.G., E.C., J.M., H.L., D.G., E.M., I.W., O.C., K.S., H.L.M. and I.C.M. contributed to acquisition of data; S.A.G., E.C., J.M., H.L., D.G., E.M., I.W., O.C., K.S., H.L.M. and I.C.M. contributed to analysis and interpretation of data; and S.A.G., E.C., H.L.M. and I.C.M were responsible for the statistical analysis. Each author contributed important intellectual content during manuscript drafting or revision and accepts accountability for the overall work by ensuring that questions pertaining to the accuracy or integrity of any portion of the work are appropriately investigated and resolved. S.A.G. and H.L.M. take responsibility that this study has been reported honestly, accurately and transparently; that no important aspects of the study have been omitted; and that any discrepancies from the study as planned and registered have been explained. The funders did not have any role in study design; collection, analysis and interpretation of data; writing the report; or the decision to submit the report for publication.

## CONFLICT OF INTEREST STATEMENT

The results presented in this article have not been published previously in whole or part.

(See related article by Johansen. Time to rehabilitate the idea of exercise for patients with chronic kidney disease? *Nephrol Dial Transplant* 2019; 34: 551--554)
