# Mortality and morbidity following exercise-based renal rehabilitation in patients with chronic kidney disease: the effect of programme completion and change in exercise capacity

**DOI:** 10.1093/ndt/gfz032

**Published:** 2019-02-15

**Authors:** Sharlene A Greenwood, Ellen Castle, Herolin Lindup, Juliet Mayes, Iain Waite, Denise Grant, Emmanuel Mangahis, Olivia Crabb, Kamer Shevket, Iain C Macdougall, Helen L MacLaughlin

*Nephrol Dial Transplant* 2018; gfy351. doi: 10.1093/ndt/gfy351

In Table 3 of the above article “Completion of RR” has been corrected to “Non-completion of RR”.

